# Distribution of *Streptococcus pneumoniae* serotypes in the northeast macro-region of São Paulo state/Brazil after the introduction of conjugate vaccine

**DOI:** 10.1186/s12879-017-2696-y

**Published:** 2017-08-25

**Authors:** Marta Inês Cazentini Medeiros, Samanta Cristine Grassi Almeida, Maria Luiza Leopoldo Silva Guerra, Paulo da Silva, Ana Maria Machado Carneiro, Denise de Andrade

**Affiliations:** 1 Regional Laboratory Center of Instituto Adolfo Lutz - Ribeirão Preto and student at College of Nursing at University of São Paulo - Ribeirão Preto, São Paulo, Brazil; 2 Bacteriologic Center of Instituto Adolfo Lutz Central–São Paulo, São Paulo, Brazil; 3 Regional Laboratory Center of Instituto Adolfo Lutz - Ribeirão Preto, São Paulo, Brazil; 4 Ribeirão Preto College of Nursing at University of São Paulo - Ribeirão Preto, São Paulo, Brazil; 5 Ribeirão Preto College of Nursing, University of São Paulo, Ribeirão Preto, São Paulo, Brazil

**Keywords:** *Streptococcus pneumoniae*, pneumococcus, serotype, conjugate vaccine

## Abstract

**Background:**

Infections caused by *Streptococcus pneumoniae* (Spn) still challenge health systems around the world, even with advances in vaccination programs. The present study evaluated the frequency of various Spn serotypes isolated in Regional Health Care Network 13 (RRAS 13), which includes the regional health departments (RHDs) of Araraquara, Barretos, Franca and Ribeirão Preto, especially after the introduction of 10-valent pneumococcal conjugate vaccine (PCV10) in 2010.

**Methods:**

The analyzed Spn strains were isolated from patients with invasive pneumococcal diseases (IPDs) and then sent to Adolfo Lutz Institute (ALI) for further confirmative identification tests during the period from 1998 to 2013. The samples were from the cities in RRAS13, which is located in the Northeast region of São Paulo State, and totals 90 municipalities.

**Results:**

We analyzed strains isolated from 796 patients. They were predominantly: men (58.9%); 20 to 60 years old (32.2%); evaluated from 2003 to 2010 (60.2%); and diagnosed with meningitis (45.7%) and pneumonia (45.0%), the most common invasive pneumococcal diseases. In 2010, serotypes 3, 19F, 1, 23F, 6A and 6B were among the most frequent, while serotypes 3, 12F, 14, 6A, 18C, 8 and 6B were more common after the introduction of PCV10. Serotypes 14, 19F and 3 were more frequent in meningitis, while serotypes 14, 3 and 1 prevailed in pneumonia. After 2010, there was a decrease in serotypes 14, 1, 23F and 5 and an increase in serotypes 3, 12F, 11A and 8, which were not present in the vaccine.

**Conclusions:**

The present study noted the increase in serotypes 3, 12F, 11A and 8 after vaccination. None of those serotypes are included in the available conjugate vaccines, which highlights the importance of continued monitoring of IPDs in order to measure the disease burden in the population in the long term and provide new epidemiological information to determine the impact of PCV10 in Brazil.

## Background^1^

*Streptococcus pneumoniae,* or pneumoccocus, represents a big threat to human health. Invasive pneumococcal diseases (IPDs) are the most serious form of pneumococcal disease, and involve pneumonia accompanied by bacteremia, meningitis, peritonitis, bacteremia, sepsis, and arthritis, among others [1]. Around 30 to 50% of pneumococcal pneumonia cases are associated with bacteremia. In the Latin American region, pneumonia is among the leading causes of hospitalization and death in children under 5 years old and the elderly over 60 years old. Bacterial meningitis is less common than pneumonia, but has a higher risk of sequelae and death [2].

Ninety-four pneumococcal serotypes have been identified, after the inclusion of serotypes 6C, 6D, 11E and 20A/20B [3–6]. They are part of the normal ecological environment of the nasopharynx, with pathogenic potential for humans when they reach normally sterile parts of the body. The serotypes are variably distributed according to region, age, and clinical syndrome over time, and they vary in virulence, invasiveness and ability to acquire drug resistance [7].

Due the increasing spread of pneumococcal resistance to antibiotics, there is heightened interest in preventing infections by using vaccination. The use of conjugate vaccines, in addition to decreasing the incidence of pneumococcal infection, reduces the consumption of antibiotics and consequently the circulation of resistant strains [8]. There are different types of pneumococcal vaccines, classified by the amount of serotypes in their composition [9]. Vaccination is unquestionably the best tool to prevent pneumococcal disease, but immunity develops specifically according to serotype [10].

Limited vaccine coverage represents a big obstacle to controlling the diseases associated with pneumococcus, as does the difficulty of including a greater number of antigens in conjugated vaccines. This means that vaccinated individuals remain susceptible to non-vaccine serotypes, which may be capable of causing diseases [10]. Therefore, there is a need to know the epidemiological profile of different communities in order to provide data to support the development of vaccines [11].

The present study aimed to describe the distribution of cases of IPDs in the Northeast region of São Paulo State according to demographic characteristics and pneumococcal serotype strains isolated from 1998 to 2013, in order to relate it to the National Vaccination Program that was implemented in 2010.

## Methods

This was a retrospective follow-up study, based on microbiological information for pneumococci isolated from individuals with IPDs during the 16-year period from 1998 to 2013. Analysis was performed on strains of *S. pneumoniae* isolated from sterile sites. The patients included in this study lived in the northeast region of São Paulo State - Brazil, in cities belonging to Regional Health Network 13 (RRAS 13), with approximately 90 municipalities, covering the Regional Health Departments (RHDs) of Araraquara, Barretos, Franca and Ribeirão Preto [12].

Microbiological information on the serotypes was collected (Serotype and antimicrobial susceptibility profile) from the database of the Regional Laboratory Center/ALI of Ribeirão Preto and the Central ALI/São Paulo.

Statistical analysis used the chi-square test between two or more independent samples to verify the dependence or independence of the variables. Thus, possible associations between the presence of specific serotypes and age, distribution of cases according to period and age, and clinical diagnosis and serotype were investigated; a *p* value of <0.05 was adopted. The Statistical Package for Social Sciences (SPSS) version 19.0 was used.

The study was approved by a Research Ethics Committee, linked to Ribeirão Preto College of Nursing, University of São Paulo and obtained permission from regional health departments for their achievement.

## Results

For the 796 strains of *S. pneumoniae* analyzed, the age range of the patients was from less than 1 month up to 93 years old (mean = 25.1, SD = 25.9, median = 13.0). A total of 58.9% of the isolates were from male patients. There was a higher number of cases in the age group of 20 to 60 years old (32.2%) and in the period from 2003 to 2010 (60.2%).

The most frequent IPDs were meningitis (45.7%) and pneumonia (45.0%), while others represented 9.3% of the cases. The RHD of Ribeirão Preto had higher occurrence of pneumonia (56.3%), differing from the other municipalities, where meningitis was more frequent (92.6% - Araraquara, 88.6% - Franca, 93.6% - Barretos). Blood was the biological material most often used for diagnosis (45.6%).

The distribution of IPDs by age changed over time, with a progressive decrease in the percentage of IPDs in individuals under 20 years old and an increase in those over 20 years old. From 1998 to 2002, the highest frequency of IPDs was observed in children under 5 years old, with a predominance in those less than 1 year old. From 2010 on, besides a decrease in the proportion of IPDs in the age groups covered by the vaccine, there was a reduction of cases in individuals under 20 years old (Table 1).

**Table 1:** Age and major invasive pneumococcal diseases (IPDs) in patients treated in Regional Health Care Network 13, according to the isolation period, 1998–2013

Age group (years)	Period						Total	
	1998–2002		2003–2010		2011–2013			
	n^o^	%*	n^o^	%*	n^o^	%*	n^o^	%
<1	51	23.5	54	11.8	7	7.4	112	14.5
1 **˫** 2	36	16.6	49	10.7	3	3.1	88	11.4
2 **˫** 5	37	17.1	52	11.3	9	9.5	98	12.7
Subtotal (<5)	124	57.1	155	33.8	19	20.0	298	38.7
5 **˫** 20	36	16.6	68	14.8	6	6.3	110	14.3
20**˫** 60	45	20.7	164	35.7	47	49.5	256	32.2
≥60	12	5.5	72	15.7	23	24.2	107	13.9
Subtotal (≥5)	93	42.9	304	66.2	76	80.0	473	61.3
Ignored^a^	5	0.6	20	2.5	0	0.0	25	31
IPD								
Meningitis	124	55.8	195	40.7	45	47.4	364	45.7
Pneumonia	79	35.6	240	50.1	39	41.0	358	45.0
Bacteremia/sepsis	7	3.2	34	7.1	8	8.4	49	6.2
Other^b^	12	5.4	10	2.1	3	3.2	25	3.1
Total	222	100.0	479	100.0	95	100.0	796	100.0

%* refers to the total IPD cases in each period

^a^Patients without information on their age

^b^Abscess, arthritis, peritonitis, pancreatitis, cirrhosis, surgical site infection and appendicitis

We found 59.4% of IPDs in individuals 5 years and older. Of these, 16.0% had meningitis. Among children under 5 years old, meningitis affected more of those under 1 year of age (8.2%) (Table 2). There was no significant change in the serotypes included in the 7-valent pneumococcal conjugated vaccine (PCV7) between 1998 and 2002 (45.9%) and 2003–2010 (48.2%) (Table 3).

**Table 2:** Age groups of patients who received care in Regional Health Care Network 13, according to main IPDs, from 1998 to 2013

Age group (years)	Diagnosis								Total	
	Meningitis		Pneumonia		Bact/sepsis		Other^b^			
	n^o^	%*	n^o^	%*	n^o^	%*	n^o^	%*	n^o^	%
<1	65	8.2	42	5.3	5	0.6	0	0.0	112	14.1
1 ˫ 2	29	3.6	54	6.8	4	0.5	1	0.1	88	11.0
2 ˫ 5	33	4.1	59	7.4	4	0.5	2	0.3	98	12.3
Subtotal < 5	127	16.0	155	19.5	13	1.6	3	0.4	298	37.4
5 ˫ 20	57	7.2	44	5.5	4	0.5	5	0.6	110	13.8
20 ˫ 60	127	16.0	101	12.7	19	2.4	9	1.1	256	32.2
≥60	42	5.3	50	6.3	9	1.1	6	0.8	107	2.1
Subtotal ≥ 5	226	28.4	195	24.5	32	4.0	20	2.5	473	59.4
Ignored^a^	11	1.4	8	1.0	4	0.5	2	0.3	25	3.1
Total	364	45.7	358	45.0	49	6.2	25	3.1	796	100.0

%* refers to the total cases

^a^Patients without information on their age

^b^Abscess, arthritis, peritonitis, pancreatitis, cirrhosis, surgical site infection and appendicitis

**Table 3:** Vaccine and non-vaccine serotypes of *S. pneumoniae* identified in patients with invasive pneumococcal disease cared for in Regional Health Care Network 13, according to the isolation period, 1998 to 2013

Serotypes	Periods						Total	
	1998–2002		2003–2010		2011–2013			
	n^o^	%*	n^o^	%*	n^o^	%*	n^o^	%
PCV7								
14	45	20.3	94	19.6	6	6.3	145	18.2
19F	15	6.8	28	5.8	3	3.2	46	5.8
6B	7	3.2	27	5.6	4	4.2	38	4.8
23F	8	3.6	29	6.1	0	0.0	37	4.6
9V	6	2.7	25	5.2	3	3.2	34	4.3
18C	13	5.9	15	3.1	5	5.3	33	4.1
4	8	3.6	13	2.7	3	3.2	24	3.0
Total PCV7	102	45.9	231	48.2	24	25.3	357	44.8
Included PCV10								
1	12	5.4	31	6.5	0	0.0	43	5.4
7 F	7	3.2	12	2.5	2	2.1	21	2.6
5	14	6.3	1	0.2	2	2.1	17	2.1
Total PCV10	33	14.9	44	9.2	4	4.2	81	10.2
Included PCV13								
3	14	6.3	42	8.8	17	17.9	73	9.2
6A	13	5.9	20	4.2	6	6.3	39	4.9
19A	13	5.9	18	3.8	2	2.1	33	4.1
Total PCV13	40	18.0	80	16.7	25	26.3	145	18.2
Non-vaccine								
12F	1	0.5	18	3.8	9	9.5	28	3.5
11A	0	0.0	12	2.5	3	3.2	15	1.9
22F	3	1.4	12	2.5	0	0.0	15	1.9
8	2	0.9	5	1.0	5	5.3	12	1.5
9N	1	0.5	6	1.3	3	3.2	10	1.3
10A	3	1.4	5	1.0	1	1.1	9	1.1
15C	2	0.9	3	0.6	4	4.2	9	1.1
17F	3	1.4	5	1.0	0	0.0	8	1.0
6C	1	0.5	3	0.6	4	4.2	8	1.0
18A	4	1.8	3	0.6	0	0.0	7	0.9
18B	2	0.9	5	1.0	0	0.0	7	0.9
23B	1	0.5	5	1.0	0	0.0	6	0.8
NT	3	1.4	3	0.6	0	0.0	6	0.8
NR	0	0.0	2	0.4	0	0.0	2	0.3
Other^a^	21	9.5	37	7.7	13	13.7	71	8.9
Total non- vaccine	47	21.2	124	25.9	42	44.2	215	27.0
Total	222	100.0	479	100.0	95	100.0	796	100.0

%* refers to the total casos of IPDs in each period, Other ^a^serotypes with less than 6 isolates

*NT* Non-typable, *NR* Not carried out (unviable strain)

PCV7 – valent pneumococcal conjugate vaccine

PCV10–10-valent pneumococcal conjugate vaccine

PCV13–13-valent pneumococcal conjugate vaccine

After the introduction of PCV10, considering the periods 2003–2010 and 2011–2013, the total number of IPDs decreased from 479 to 95, associated mainly with the reduction of vaccine serotypes. The proportion of serotypes included in 13-valent pneumococcal conjugate vaccine (PCV13) increased from 2003 to 2010 to 2011–2013, as well as non-vaccine serotypes 12F, 11A, 8, 9 N, 15C and 6C. From 1998 to 2010, serotype 14 was the most prevalent, but after 2010 this serotype decreased from 19.6 to 6.3% (Table 3).

Fifty-four pneumococcus serotypes were identified. Serotypes 14, 3, 19F, 1, 6A, 6B, 23F, 9 V, 18C, 19A, 12F, 4, 7F, 5, 11A, 22F, 8 and 9 N represented 83.3% of the cases. Serotype 14 was the most common serotype in the four RHDs studied. In descending order, serotypes 3, 1, 19F, 6A, 6B, 19A, 23F and 9 V were the most isolated in the RHD of Ribeirão Preto; 3, 18C, 19F, 9 V, 11A, 6A, 17F and 23F in the RHD of Araraquara; 23F, 3, 19F, 22F, 6A, 6B and 7F in the RHD of Franca; and 6B, 3, 19F, 1, 18C, 19A and 23F in the RHD of Barretos. Note that serotypes 14, 3, 19F and 23F were the most frequent in the four RHDs.

In the period preceding the introduction of PCV10, there was predominance, in descending order, of serotypes 14, 3, 19F, 1, 23F, 6B and 6A. After the introduction of vaccination, there was a predominance of serotypes 3, 12F, 14, 6A, 18C, 8 and 6B. Up to 2010, serotype 14 was the most frequent, with a significant decrease in the last studied period; it was surpassed by serotype 3 after 2011. Evaluating all age groups and serotypes with 10 or more isolates, there was a statistically significant decrease in serotypes 14, 1, 23F and 5 and increase in serotypes 3, 12F, 11A and 8 (Table 4).

**Table 4:** Main serotypes of *S. pneumoniae* isolated from patients with invasive pneumococcal disease cared for in Regional Health Care Network 13, according to the isolation period,1998 to 2013

Serotypes	1998–2002 *n* = 222		Period						Total		*p* value
			2003–2010 *n* = 479		Subtotal *n* = 701		2011–2013 *n* = 95		*n* = 796		
	n^o^	%*	n^o^	%*	n^o^	%	n^o^	%*	n^o^	%	
14	45	20.3	94	19.6	139	19.8	6	6.3	145	18.2	**0.006**
3	14	6.3	42	8.8	56	8.0	17	17.9	73	9.2	**0.004**
19F	15	6.8	28	5.8	43	6.1	3	3.2	46	5.8	0.451
1	12	5.4	31	6.5	43	6.1	0	0.0	43	5.4	**0.039**
6A	13	5.9	20	4.2	33	4.7	6	6.3	39	4.9	0.501
6B	7	3.2	27	5.6	34	4.9	4	4.2	38	4.8	0.344
23F	8	3.6	29	6.1	37	5.3	0	0.0	37	4.6	**0.026**
9V	6	2.7	25	5.2	31	4.4	3	3.2	34	4.3	0.262
18C	13	5.9	15	3.1	28	4.0	5	5.3	33	4.1	0.205
19A	13	5.9	18	3.8	31	4.4	2	2.1	33	4.1	0.245
12F	1	0.5	18	3.8	19	2.7	9	9.5	28	3.5	**<0.001**
4	8	3.6	13	2.7	21	3.0	3	3.2	24	3.0	0.811
7F	7	3.2	12	2.5	19	2.7	2	2.1	21	2.6	0.832
5	14	6.3	1	0.2	15	2.1	2	2.1	17	2.1	**<0.001**
11A	0	0.0	12	2.5	12	1.7	3	3.2	15	1.9	**0.047**
22F	3	1.4	12	2.5	15	2.1	0	0.0	15	1.9	0.206
8	2	0.9	5	1.0	7	1.0	5	5.3	12	1.5	**0.006**
9N	1	0.5	6	1.3	7	1.0	3	3.2	10	1.3	0.140

%* refers to the total IPD cases in each period

*p* values in bold are significant

Serotypes 14 and 3 were the most often associated with meningitis and pneumonia. Serotype 1 was not detected among the main causes of meningitis (Table 5). Serotype 1 greatly affected patients from 2 to 20 years old (Table 6).

**Table 5:** *S. pneumoniae* serotypes isolated from patients with invasive pneumococcal disease cared for in Regional Health Care Network 13 according to the main IPDs diagnosed, 1998 to 2013

Serotype	Meningitis		Pneumonia		Bact/sepsis		Other		Total	
	n^o^	%*	n^o^	%*	n^o^	%*	n^o^	%*	n^o^	%*
14	45	5.7	95	11.9	3	0.4	2	0.3	145	18.2
3	34	4.3	35	4.4	4	0.5	0	0.0	73	9.2
19F	30	3.8	12	1.5	3	0.4	1	0.1	46	5.8
1	7	0.9	33	4.1	1	0.1	2	0.3	43	5.4
6A	22	2.8	12	1.5	0	0.0	5	0.6	39	4.9
6B	15	1.9	19	2.4	3	0.4	1	0.1	38	4.8
23F	16	2.0	16	2.0	3	0.4	2	0.3	37	4.6
9V	19	2.4	12	1.5	3	0.4	0	0.0	34	4.3
18C	22	2.8	8	1.0	2	0.3	1	0.1	33	4.1
19A	15	1.9	14	1.8	3	0.4	1	0.1	33	4.1
12F	21	2.6	4	0.5	2	0.3	1	0.1	28	3.5
4	7	0.9	14	1.8	2	0.3	1	0.1	24	3.0
7F	8	1.0	9	1.1	2	0.3	2	0.3	21	2.6
5	4	0.5	12	1.5	0	0.0	1	0.1	17	2.1
11A	10	1.3	5	0.6	0	0.0	0	0.0	15	1.9
22F	8	1.0	7	0.9	0	0.0	0	0.0	15	1.9
8	4	0.5	6	0.8	2	0.3	0	0.0	12	1.5
9N	2	0.3	5	0.6	3	0.4	0	0.0	10	1.3
Other	75	9.4	40	5.0	13	1.6	5	0.6	133	16.7
Total	364	45.7	358	45.0	49	6.2	25	3.1	796	100.0

%* refers to the total cases

**Table 6:** Main *S. pneumoniae* serotypes isolated from patients with invasive pneumococcal disease cared for in Regional Health Care Network 13, according to age, from 1998 to 2013

Serotype		Age group (years)															Total		*p* value
	< 1 *n* = 112		1˫ 2 *n* = 88		2˫5 *n* = 98		Subtotal < 5 n = 298		5˫20 *n* = 110		20˫60 *n* = 256		≥60 *n* = 107		Subtotal ≥ 5 *n* = 473		*n* = 771		
	n^o^	%*	n^o^	%*	n^o^	%	n^o^	%*	n^o^	%*	n^o^	%*	n^o^	%*	n^o^	%	n^o^	%	
14	37	33.0	36	40.9	38	38.8	**111**	**37.2**	8	7.3	15	5.9	7	6.5	**30**	**6.3**	141	18.3	**<0.001**
3	8	7.1	5	5.7	3	3.1	**16**	**5.4**	5	4.5	34	13.3	17	15.9	**56**	**11.8**	72	9.3	**0.001**
19F	3	2.7	1	1.1	5	5.1	**9**	**3.0**	11	10.0	13	5.1	9	8.4	**33**	**7.0**	42	5.4	**0.005**
1	3	2.7	2	2.3	11	11.2	**16**	**5.4**	16	14.5	7	2.7	3	2.8	**26**	**5.5**	42	5.4	**<0.001**
6A	10	8.9	3	3.4	8	8.2	**21**	**7.0**	7	6.4	9	3.5	2	1.9	**18**	**3.8**	39	5.1	**0.079**
6B	6	5.4	10	11.4	3	3.1	**19**	**6.4**	0	0.0	13	5.1	5	4.7	**18**	**3.8**	37	4.8	**0.012**
23F	4	3.6	5	5.7	2	2.0	**11**	**3.7**	6	5.5	12	4.7	7	6.5	**25**	**5.3**	36	4.7	0.696
9V	4	3.6	4	4.5	3	3.1	**11**	**3.7**	5	4.5	8	3.1	10	9.3	**23**	**4.9**	34	4.4	0.167
18C	6	5.4	4	4.5	6	6.1	**16**	**5.4**	8	7.3	5	2.0	3	2.8	**16**	**3.4**	32	4.2	0.174
19A	10	8.9	3	3.4	3	3.1	**16**	**5.4**	2	1.8	9	3.5	6	5.6	**17**	**3.6**	33	4.3	0.123
12F	1	0.9	1	1.1	1	1.0	**3**	**1.0**	4	3.6	15	5.9	4	3.7	**23**	**4.9**	26	3.4	**0.074**
4	0	0.0	0	0.0	2	2.0	**2**	**0.7**	2	1.8	16	6.2	4	3.7	**22**	**4.7**	24	3.1	**0.007**
7F	2	1.8	2	2.3	1	1.0	**5**	**1.7**	3	2.7	9	3.5	3	2.8	**15**	**3.2**	20	2.6	0.825
5	2	1.8	3	3.4	1	1.0	**6**	**2.0**	3	2.7	6	2.3	0	0.0	**9**	**1.9**	15	1.9	0.531
11A	0	0.0	0	0.0	0	0.0	**0**	**0.0**	2	1.8	10	3.9	2	1.9	**14**	**3.0**	14	1.8	**0.036**
22F	1	0.9	0	0.0	1	1.0	**2**	**0.7**	1	0.9	7	2.7	3	2.8	**11**	**2.3**	13	1.7	0.404
8	1	0.9	0	0.0	0	0.0	**1**	**0.3**	0	0.0	9	3.5	1	0.9	**10**	**2.1**	11	1.4	**0.027**
9N	0	0.0	1	1.1	0	0.0	**1**	**0.3**	3	2.7	5	2.0	1	0.9	**9**	**1.9**	10	1.3	0.357

%* refers to the total number of cases in each age group - 25 patients were excluded for lack of information regarding the age range

*p* values in bold are significant

Serotype 3 occurred mostly in adults. However, its importance was also noted in children under 2 years old. In children under 5 years old, 83% of the isolates were of serotypes 14, 6A, 6B, 3, 1, 18C, 19A, 23F, 19F and 9 V. In children over 5 years of age, 71% of pneumococci were related to serotypes 3, 19F, 14, 1, 23F, 9 V, 12F, 4, 6A, 6B, 19A, 18C, 7F and 11A (Table 6).

During the study, prevalence of serotype 19A did not change, being more isolated in children under 5 years of age, especially in those less than 1 year old. Serotypes 14, 6B, 6A, 19A and 3 were among the main causes of IPDs in children under 2 years old. In the age group of 60 years old or older, serotypes 3, 9 V, 19F and 23F were the most common (Table 6).

Considering the most common serotypes and cross-immunity between 6A and 6B, the estimated vaccine serotypes isolated from children under 5 years old reached 69.2% for PCV10 and 87.0% for PCV13. However, for those over 5 years old, the percentage of serotypes present in PCV10 and PCV13 was 46.0% and 65.2%, respectively. In the analyzed period, serotypes 6A, 3 and 19A represented 17.8%, 15.9 and 19.2%, respectively, in patients younger than 5 years old and older than 5 years old (Table 6).

## Discussion

In the present study, blood was the biological material most often used for diagnosis. The isolation of pneumococcus in culture is considered the gold standard for the definition of pneumococcal disease, although the positivity of blood culture is low, especially in children [13]. It is worth highlighting the importance of routine blood culture for patients suspected of having bacteremia, particularly in relation to the meningitis diagnosis, in which blood culture is often neglected when just the culture of cerebrospinal fluid is targeted [14].

Besides the low positivity of blood culture, another aggravating factor for proper diagnosis, especially for pneumonia, is cases that are cared for in clinics and treated empirically, without collection of biological samples for identification of microorganisms. Diagnosis is also hampered by previous use of antibiotics before the collection of biological material [15]. It is estimated that around 50% of cases of pneumonia remain undiagnosed [16]. Therefore, the extent of pneumococcal disease is underestimated and difficult to assess.

In the present study, one possible reason for the higher frequency of meningitis is its compulsory notification and a more active surveillance system when compared to pneumonia.

After the introduction of PCV7 in the United States, beyond the direct effect on the reduction of cases of pneumococcal disease in the vaccinated population, the herd effect was also observed, which acted indirectly to prevent disease in non-vaccinated individuals as a consequence of reduced circulation of vaccine serotypes [10]. The data presented in the current study were through 2013, so a further observation period in the unvaccinated population would be necessary to detect the full effect of herd immunity.

In Brazil, PCV7 was introduced in 2002, and was provided free of charge only to people at high risk of acquiring IPD and in private clinics, reaching a small portion of the population, verifying the absence of response to this intervention.

In 2010, Brazil introduced PCV10 into its routine National Immunization Program, aiming to minimize the impact of pneumococcal disease. Research on the effectiveness of PCV10 implementation for public health services in Brazil has indicated a reduction in children’s hospitalizations for pneumonia [17]; protection of nasopharyngeal pneumococcal carriers against vaccine serotypes [18]; and reduction of IPDs cases in all age groups, especially in children under 2 years old [19].

In 2011 and 2012, vaccination coverage of PCV10 in Brazil was 82.1% and 88.4%, respectively [20]. In the cities of Ribeirão Preto, Araraquara, Franca and Barretos, the coverage of PCV10 in children under 5 years old was around 86% [21], not reaching the ideal rate of 95% recommended by the Ministry of Health for efficient vaccination coverage for PCV10. In addition, completion of 3 doses of the vaccine was necessary in order to ensure the reduction of IPD cases [22], which explains the persistence after 2010 of some serotypes contained in the vaccine.

Classically, pneumococcal diseases mainly affect children and the elderly [1]. However, an understanding of pneumococcal epidemiology should involve all age groups. In mid-2012 in Argentina and Bolivia, most cases of IPDs occurred in patients between 2 and 5 years old. In Brazil, there was a higher incidence in people between 30 and 49 years old [23], coinciding with our data.

Meningitis is commonly associated with children under 2 years old, and mostly under 1 year old [14]. Contradicting our data, in Uberlândia, the Federal District [24] and Rio Grande do Norte [25], pneumococcal meningitis prevailed in children under 5 years old. In the region studied, meningitis occurred mainly in adults.

From 2001 to 2011 in Brazil, 282,593 cases of meningitis were identified; 100,559 of those were bacterial meningitis, of which 13,469 were related to *S. pneumoniae* [14]. From 1999 to 2010, it was observed that 32.5% of bacterial meningitis in Rio Grande do Sul was caused by pneumococcus [26]. In the present study, the percentage of patients with meningitis was higher than that.

Considering that in Brazil, PCV10 is given free only to children under 2 years old, the occurrence of IPDs in patients over 5 years old is relevant information, especially taking into account that in recent decades, increases in the elderly Brazilian population have accelerated [27]. This highlights the importance of constant surveillance, aiming to detect changes in the circulation of serotypes in this age group.

With the use of conjugate vaccines, a decrease in vaccine serotypes, and an increase in those not included in the vaccines, a phenomenon known as serotype replacement has been observed in several countries [28–33]. Therefore, when evaluating the effect of pneumococcal vaccines, the overall rate of disease occurrence may not decrease significantly, because of an increase in other non-vaccine serotypes [29].

The increase in non-vaccine serotypes detected in the present study suggests serotype replacement. This conflicts with a study done in São Paulo that showed no significant increase in non-vaccine serotypes in IPDs after the introduction of PCV10 [19]. Fluctuations in the occurrence of serotypes can happen naturally or by selective pressure caused by vaccines, so increases in non vaccine serotypes should be carefully evaluated. Complexes and varied factors are involved in the replacement phenomenon, making it difficult to analyze [34].

Currently, the most advanced technology for pneumococcal vaccine is polysaccharides containing capsular conjugate vaccines linked to a carrier protein. However, there have been studies of the implementation of a new generation of vaccines based on common protein components of *S. pneumoniae* that provide serotype independent immunity [35]. According to the World Health Organization, adoption of this new technology will need to provide equivalent or greater benefit than that obtained with conjugate vaccines to prevent pneumococcal disease [36].

Thus, changes in the incidence of pneumococcal serotypes associated with the disease after the use of conjugate vaccines must be distinguished from normal serotype temporal changes [1].

In this scenario, serotype replacement after vaccination may raise questions regarding the effect of this intervention. However, some non-vaccine serotypes seem to be less invasive than those present in the vaccine, so that reduction of IPDs becomes a more positive effect than eventual serotype replacement [37].

The target for prevention of pneumococcal diseases with vaccines is the bacterial capsule. The effect of vaccination can be reduced by the classical phenomenon of capsular exchange, in which a vaccine serotype changes its capsular locus and begins to express other non-vaccine serotypes [38].

Noteworthy is an increase in serotypes 3, 12F, 11A and 8, reinforcing the need for continued vigilance. With the exception of Serotype 3 included in PCV13, no other serotypes are included in the available vaccine formulations (PCV10 and PCV13).

An example of serotype replacement is what happened with 19A, which is cited as one of the emerging serotypes in the United States since the introduction of PCV7 [30]. However, it stabilized after an increase in the use of PCV13, in which it is included [39]. During the present study, prevalence of serotype 19A did not change, which is similar to what was reported in São Paulo [19]. There was a predominance of 19A in children under 5 years old. This is similar to results found in the United States, where the frequency of this serotype in children under 6 years old was substantial [40].

In Brazil, as in the Caribbean and some other Latin American countries, there is low frequency of serotype 19A [2]. Although a case-control study on the impact of PCV10 in Brazil showed cross-protection between serotypes 19F and 19A [41], an increase in the circulation of Serotype 19A was reported in 2012 [23].

In the Central Laboratory of Paraná, in 2001–2002, pneumococcus was the most common etiologic agent in acute bacterial meningitis, with higher incidence of serotypes 14, 23F and 3 [42]. In our study, serotypes 14, 3 and 19F were more involved in meningitis. The potential for invasion expressed by Serotype 1 has been linked to outbreaks and fatal cases of meningitis in Africa [43] and in some countries in Europe [44], but in the present study, Serotype 1 was not detected among the main causes of meningitis. In Latin America, this serotype has been considered a major cause of IPDs in all ages [45].

Serotype 3 has a specific virulence factor for pneumonia associated with severe disease cases [46], especially in the elderly [47]. This corroborates the present study, in which this serotype was important in pneumonia.

In RRAS 13, serotypes 14, 6B, 6A, 19A and 3 were among the leading causes of IPDs in children younger than 2 years old. Serotypes 14 and 6B are included in PCV10; the remaining serotypes are included only in PCV13. In 2010, countries like Italy [48] and the U.S. [49] replaced PCV7, in use since 2000, with PCV13 because of its higher serotype coverage.

In the age group of 60 years old or older, serotypes 3, 9 V, 19F and 23F were the most common. One study found that in Argentina, most IPDs were caused by serotypes 14, 1 and 5, and in Bolivia the most frequent serotypes were 14, 6B and 18F [23].

Among the specific serotypes of PCV13, serotypes 6A and 19A did not present a significant increase during the course of the present study. Serotype 3 presented a progressive increase (Tables 3 and 4), especially in cases of meningitis and pneumonia (Table 5), with emphasis on patients over 20 years old (Table 6). This increase should be monitored to evaluate the circulation of this serotype in the community.

This research has limitations. Because it is a retrospective study, results and conclusions are based on social and microbiological pre-existing information, thus are subject to information bias. In addtion, there is to consider the use of sampling related to convenience criterion adopted for the study.

## Conclusions

After the introduction of PCV10, there was a decrease in the overall percentage of vaccine serotypes and an increase in some non-PCV10 serotypes. Of note is the increase in serotypes 3, 12F, 11A and 8 after vaccination. Considering that 12F, 11A, and 8 are not included in the available conjugate vaccines, and the possibility of serotype replacement after the use of conjugated vaccines, it is necessary to implement studies of new vaccine generation based on the common protein component of *S. pneumoniae*, which is capable of providing serotype-independent immunity. The finding of a high percentage of IPDs patients in the 20 to 60 age group highlights the importance of continuous monitoring of IPDs to assess the long-term disease burden in the population, especially in adult patients, who are not routinely covered by vaccination. Such monitoring would provide new epidemiological information to determine the impact of PCV10 in Brazil. Due to the significant increase in Serotype 3 found in the present study, the use of PCV13 as an alternative to PCV10 in public health services in Brazil could reduce the percentage of IPDs in the studied region.

## Abbreviations

ALI: Adolfo Lutz Institute
IPD: Invasive pneumococcal disease
OPAS: Pan American Health Organization
PCV: Valent pneumococcal conjugate vaccine
RHD: Regional health department
RRAS: Regional Health Care Network
SD: standard deviation
Spn: *S. pneumoniae*
SPSS: Statistical Package for Social Sciences
WHO: World Health Organization

## Footnotes

1 Non-standard abbreviations: Regional Healthcare Network 13 (RRAS13); regional health departments (RHDs); Adolfo Lutz Institute (ALI).
